# The Metropolitan Hospital Sunday Fund and Letters of Recommendation

**Published:** 1907-01-05

**Authors:** 


### THE METROPOLITAN HOSPITAL SUNDAY FUND AND LETTERS OF RECOMMENDATION.

A HOSPITAL OFFICIAL writes: But a passing note was made in the last issue of THE HOSPITAL to the remarks of Mr. Henry Morris, who at the annual meeting of the Metropolitan Hospital Fund, asked permission to point out what he thought were two blots on the working of the Fund. One was that the letters of recommendation issued by hospitals to the Fund were dated, and were not accepted after expiry, and the other was that private subscribers were accorded twice as many "letters" as the Fund for each unit of subscription.

In regard to the first point there are very solid reasons why letters of recommendation should be dated. In the first place an annual subscriber's privileges are everywhere confined to the year for which the subscription is paid. To quote but one instance, no member of a club would claim for the privileges of his club to be extended to him over a second year for the same subscription, should he not have used the club during the year for which the subscription was paid. The effect of this limitation in regard to hospitals is that it regularises the work of the hospitals. If "letters" were to be honoured at any time, hospitals would be liable to have at one season large numbers of patients awaiting admission, and then at another, because all "letters" have been presented, no patients applying and numbers of beds vacant. There is now a great possibility of this in special hospitals and special departments, due to the great changes in some forms of treatment, particularly electrical treatment and in apparatus. For instance, the skin hospitals and the skin departments of the general hospitals during the early part of 1906 were thronged with cases of ringworm owing to the discovery that the complaint could be cured in twenty minutes by application of $x$-rays, as against the weeks, and sometimes months, required by medicinal treatment.

Then, again, a few wealthy Governors with an accumulated number of "letters" might swamp the hospital with applications and exclude the patients recommended by a large number of individual Governors. This would have a very disastrous effect upon the subscription list, for the hospital would lose a large amount in new annual subscriptions from persons desirous of securing the early admission of cases in which they were personally interested and who otherwise would not subscribe.

Then, quite apart from these considerations, the particulars of procedure, times of attendance, terms of admission, etc., are modified from time to time, and the "letters" thus become out of date, and by their presentation endless trouble, confusion, and disappointment, would be caused to patients, and to those coming long distances the matter would be serious. As at present arranged, alterations of this character are deferred until the next issue of "letters."

With regard to the second point, it must be remembered that primarily the object of the Hospital Sunday Fund is to assist the hospitals, and if the Fund required no privileges the grants would represent gifts to the hospitals of the face value of the awards. The Council, however, doubtless recognising the principle underlying the very system of "letters," that human nature requires a *quid pro quo*, requests hospitals issuing letters of recommendation to send supplies of them for the Fund to issue at its discretion to the contributing clergy, and this completely alters the situation. The Fund may make a grant of £100 to a particular hospital and yet take from the hospital treatment for patients to the value of £200. To explain this, the following instance may be quoted. Suppose a special hospital to give one in-patient "letter" for an annual subscription of one guinea, the average period of residence to be three months and the average cost per occupied bed £85 per annum. These are by no means extreme figures. Then for an annual subscription of one guinea the Governor receives treatment for his patient to the value of £20. He takes out of the hospital twenty times what he pays in! Small wonder then that many hospitals are badly in need of funds. The privileges accorded to Governors are far too generous in the great majority of cases.

It is, of course, possible to give Governors a privilege that is more in treatment than is covered by their subscriptions, because experience shows that Governors do not use all their "letters" every year, but the Hospital Sunday Fund is not on the same footing as a private Governor, and although a number of the recommendations issued to the Hospital Sunday Fund are not used, yet the large number that are must be a great tax upon the resources of the hospitals.

Of course the effect of ceasing to issue "letters" to the Fund would undoubtedly be a drop in the amount of the awards, but the parishioners who had been in the habit of obtaining the necessary "letters" from the clergy would be obliged to subscribe to the hospitals for which they needed recommendations, and the hospitals would probably be the gainers in the long run.

---

PROFESSOR HALLIBURTON, F.R.C.P., will deliver an address on "The Diet of To-day," at 34 Devonshire Street, W., on January 9, at 4 P.M.

